# Perceptions of HIV-Related Comorbidities and Usability of a Virtual Environment for Cardiovascular Disease Prevention Education in Sexual Minority Men With HIV: Formative Phases of a Pilot Randomized Controlled Trial

**DOI:** 10.2196/57351

**Published:** 2024-08-22

**Authors:** S Raquel Ramos, Harmony Reynolds, Constance Johnson, Gail Melkus, Trace Kershaw, Julian F Thayer, Allison Vorderstrasse

**Affiliations:** 1 School of Nursing Yale University Orange, CT United States; 2 School of Public Health Social and Behavioral Sciences Yale University New Haven, CT United States; 3 Center for Interdisciplinary Research on AIDS Yale University New Haven, NY United States; 4 Cardiovascular Clinical Research Center Leon H. Charney Division of Cardiology, Department of Medicine NYU Grossman School of Medicine New York, NY United States; 5 Czik School of Nursing The University of Texas Health Science Center at Houston Houston, TX United States; 6 McWilliams School of Biomedical Informatics The University of Texas Health Science Center at Houston Houston, TX United States; 7 Rory Myers College of Nursing New York University New York, NY United States; 8 School of Social Ecology, Psychological Science University of California Irvine, CA United States; 9 Elaine Marieb College of Nursing University of Massachusetts Amherst Amherst, MA United States

**Keywords:** virtual environment, digital health, gamification, eHealth, sexual minorities, cardiovascular disease, HIV, cardiometabolic risk, mental health, lesbian, gay, bisexual, transgender, and queer, LGBTQ health, HIV care, prevention, virtual, minority, men, Latin, Black, men who have sex with men, intervention, high blood pressure, myocardial infarction, preventive health screenings, gay, bisexual, patients, cancer

## Abstract

**Background:**

Sexual minority men with HIV are at an increased risk of cardiovascular disease (CVD) and have been underrepresented in behavioral research and clinical trials.

**Objective:**

This study aims to explore perceptions of HIV-related comorbidities and assess the interest in and usability of a virtual environment for CVD prevention education in Black and Latinx sexual minority men with HIV.

**Methods:**

This is a 3-phase pilot behavioral randomized controlled trial. We report on formative phases 1 and 2 that informed virtual environment content and features using qualitative interviews, usability testing, and beta testing with a total of 25 individuals. In phase 1, a total of 15 participants completed interviews exploring HIV-related illnesses of concern that would be used to tailor the virtual environment. In phase 2, usability testing and beta testing were conducted with 10 participants to assess interest, features, and content.

**Results:**

In phase 1, we found that CVD risk factors included high blood pressure, myocardial infarction, stroke, and diabetes. Cancer (prostate, colon, and others) was a common concern, as were mental health conditions. In phase 2, all participants completed the 12-item usability checklist with favorable feedback within 30 to 60 minutes. Beta-testing interviews suggested (1) mixed perceptions of health and HIV, (2) high risk for comorbid conditions, (3) virtual environment features were promising, and (4) the need for diverse avatar representations.

**Conclusions:**

We identified several comorbid conditions of concern, and findings carry significant implications for mitigating barriers to preventive health screenings, given the shared risk factors between HIV and related comorbidities. Highly rated aspects of the virtual environment were anonymity; meeting others with HIV who identify as gay or bisexual; validating lesbian, gay, bisexual, transgender, queer, and others (LGBTQ+) images and content; and accessibility to CVD prevention education. Critical end-user feedback from beta testing suggested more options for avatar customization in skin, hair, and body representation. Our next phase will test the virtual environment as a new approach to advancing cardiovascular health equity in ethnic and racial sexual minority men with HIV.

**Trial Registration:**

ClinicalTrials.gov NCT04061915; https://clinicaltrials.gov/study/NCT05242952

**International Registered Report Identifier (IRRID):**

RR2-10.2196/38348

## Introduction

### Background

In the United States, cardiovascular disease (CVD) has been the leading contributor to mortality for >100 years [1]. Heart disease risk is especially prevalent among persons with HIV [2-4]. Antiretroviral therapy has increased life expectancy, permitting the link between HIV and premature-onset CVD to come to light. Persons with HIV are 1.5 to 2 times more likely to develop CVD than persons without HIV [4]. This increase in CVD risk is similar to the increase in CVD risk due to diabetes [4,5]. It is estimated that 78% of persons with HIV will have CVD by the year 2030 [6,7].

The risk of HIV, as well as CVD as a comorbidity, disproportionately impacts Black and Latinx sexual minority men [8], as they experience higher rates of HIV-related conditions, such as high blood pressure and diabetes [9]. CVD risk is intersectional and exacerbated by social determinants and structural forces. When these multilevel stressors coalesce, they create barriers to education, safe neighborhoods, resources, and quality health care for those who are economically, racially, and ethnically marginalized. In sexual minoritized individuals, this is intensified and manifests in the forms of repeated experiences of racism, discrimination, homophobia, and violence [10,11]. CVD risk is heightened by racism and discrimination [10,11] and their persistent effects on allostatic load [12] and weathering [13] (the ongoing, cumulative burden of stress that predisposes individuals to morbidity and mortality). Allostatic load has adverse impacts on the immune, cardiovascular, and metabolic systems over time [14], which can increase stress responses and activate the hypothalamic-pituitary axis. This creates an increase in cortisol and other inflammatory markers, resulting in physiological disruptions [15], leading to cardiometabolic and mental health morbidities [16]. Thayer et al [10] and Hill and Thayer [17] identified a “cardiovascular conundrum,” in which perpetual discrimination in Black and sexual minoritized individuals leads to both elevated systemic vascular resistance, which can heighten CVD morbidity and mortality risk, and higher vagally mediated heart rate variability (HRV), typically associated with lower risk. Exposure to discrimination and racism lowers HRV and may elicit maladaptive coping mechanisms, such as rumination, which further lowers HRV [17-19]. Racism and discrimination toward racial, ethnic, and sexual minoritized individuals have resulted in longstanding disadvantages in achieving health equity. These are root causes of medical mistrust, are catalysts for avoiding care engagement [20], and reduce opportunities for CVD screening and preventive counseling [21]. A review examining nonpharmacologic behavioral interventions to prevent CVD in persons with HIV suggested that over the past decade, there has been an increase in behavioral CVD interventions [22]. However, the extant literature is limited in its representation of populations that continue to be disproportionately affected by low cardiovascular health [22]. Current research also identified the interplay among an individual’s race, gender, and sexual orientation [23] that negatively affects health outcomes and further increases the risk of chronic conditions [24], creating an urgent need to mitigate health risks in disadvantaged, underserved, and underresourced populations. Clinical research on behavioral and lifestyle interventions to prevent heart disease that are culturally salient and tailored is essential to mitigating CVD disparities in minoritized populations. The standard approach to prevention education has been conventional patient teaching within the clinical setting. However, the literature confirms the existence of an equity gap in who has access to timely [25], quality care [26] and who has access to participation in clinical trials [27,28]. The health care and research enterprise has rapidly shifted toward ubiquitous computing as a common source of data collection for research (eg, electronic health records, artificial intelligence, wearables, mobile health, data processing via virtual environments, and augmented reality) [29-34]. Behavioral research using virtual environments and augmented reality has been gaining popularity [35-38]. However, the level of accessibility to this type of research has remained limited in diverse and marginalized populations. The novelty and relevance of technology-driven behavioral research have not been fully realized in low-income and urban communities where large numbers of minoritized individuals reside.

### This Study

The American Heart Association has called for an increase in interventions that focus on enhancing cardiovascular health in underserved communities by addressing social determinants and modifiable risk factors of CVD [39]. Specifically, the American Heart Association has called for more cardiovascular health interventions to be taken from the clinical setting and implemented into the community and community-serving programs. Community-based interventions are sought for their value in engaging individuals and families from disadvantaged or marginalized groups [40]. This provides an exceptional opportunity to bridge the equity gap that currently exists—the reason why clinical interventions do not reach marginalized individuals at increased risk. To mitigate longstanding barriers to health equity and advance understanding about the social determinates that impede cardiovascular health and perceptions of other HIV-related comorbidities of concern, qualitative, community-engaged approaches are needed. This study aimed to explore perceptions of HIV-related comorbidities and assess the usability and functionality of a virtual environment for CVD prevention education in Black and Latinx sexual minority men with HIV.

## Methods

### Study Design

This study is a 3-phase pilot behavioral randomized controlled trial. The purpose of the intervention is to test the acceptability and feasibility of a virtual environment for CVD prevention education in Black and Latinx sexual minority men with HIV using the Diabetes Learning in a Virtual Environment (LIVE) platform [35,37]. The protocol for the study is reported elsewhere [33] and is registered on Clinicaltrials.gov (NCT04061915). Formative phases 1 and 2 informed virtual environment content and features using qualitative interviews, usability testing, and beta testing with a total of 25 individuals. This paper reports on phases 1 and 2 that were necessary to inform the phase-3 clinical trial.

### Participants

We wanted to better understand chronic illness health needs from the perspectives of both HIV health experts and individuals with HIV. We partnered with 2 community-based organizations that provide supportive services to ethnic, racial, immigrant, and sexual minoritized individuals. The organizations were located in New York City and served individuals who were economically marginalized. We refer to “clients” of the community-based organizations as study “participants.”

We engaged several experts in HIV care with clinical and community health backgrounds. Eligibility criteria for the HIV care experts were as follows: (1) engagement in health care or care services with persons living with HIV for at least 5 years, (2) any gender, and (3) age >18 years.

Client participants were all adults and identified as being of either Latinx or Black heritage. Eligibility criteria for participants were as follows: (1) self-identification as gay or bisexual, (2) HIV serostatus positive, (3) ages 30 to 65 years, and (4) access to a laptop or desktop. Participants were ineligible if they (1) identified as heterosexual; (2) were HIV negative; or (3) had a history of myocardial infarction, stroke, or cognitive impairment.

### Recruitment

Before recruitment, we convened with administrators at the community-based organizations to discuss study objectives and approaches to data collection, describe the types of data that would be collected, and outline logistics for obtaining the data in ways that would be comfortable for the participants. Program managers and administrators from the community-serving organizations posted flyers and used word of mouth to provide information about the study. We worked with the community-serving organizations to contact individuals who conveyed interest in learning more about the study. Using Zoom (Zoom Video Communications), interested persons were screened, provided consent (if eligible), and were enrolled.

### Ethical Considerations

This study was approved by the Yale University Institutional Review Board (2000031403) in 2022, the New York University Institutional Review Board (IRB-FY2018-2284) in 2019, and the University of Texas Health Science Center at Houston (HSC-SN-20-1143) in 2020. All procedures performed in this study involving human participants are in accordance with the ethical standards of the institutional and national research committees, the 1964 Helsinki Declaration and its later amendments, or comparable ethical standards. All study data were deidentified to protect the identity of participants. Verbal informed consent was obtained from all individuals who participated in the study. We provided a US $30 gift card as compensation for participation.

### Study Procedures

#### Overview

Phases 1 and 2 were conducted using Zoom. Zoom is a Health Insurance Portability and Accountability Act–compliant, web-based videoconferencing platform that uses end-to-end encryption. During the interviews and focus groups, cameras were turned off. Only audio recordings using pseudonyms were used during data collection.

#### Phase 1: Qualitative Interviews

Phase-1 data were collected from June 2021 to May 2022 with a total of 15 participants. The purpose of phase 1 was to learn about health concerns that were important to participants and use this information to tailor the content and design of the virtual environment, which would be piloted in phase 3. Our interview questions were derived from an open-ended interview guide. The interview guide included 5 questions with probes for clarity. An example question is as follows: “Tell me about any medical conditions, other than HIV, that you are concerned about?” Example questions from the guide were reviewed with the program managers at the community-serving organizations to ensure that the language and content were appropriate for their clients. To ensure participant accessibility and convenience, semistructured interviews and focus groups (for participants who preferred to have an in-person, group interview at their community-serving organization) were conducted. Before starting the focus groups, ground rules were established for respect and the confidentiality of the information to be shared. The first author (SRR), an academic researcher, had a preexisting relationship with the community-based organizations and facilitated qualitative data collection. For both semistructured interviews and focus groups, pseudonyms were created before the interviews to maintain anonymity. After the interviews were completed, we used the data to embed the virtual environment with content that was reported as important, relevant, and culturally salient.

#### Phase 2: Usability and Beta Testing

After we had embedded the virtual environment with content from phase 1, we conducted usability and beta testing. Data for phase 2 were collected from August 2022 to October 2022 with a total of 10 participants. A 12-item usability checklist (Textbox 1) was developed and pilot-tested by members of the study team. The purpose of the usability checklist was to conduct a task analysis to assess the practical functionality of the virtual environment.

Example checklist items include the following: (1) “customize your avatars hair, skin, clothes, and shoes” and (2) “use the microphone to say hello to others in the virtual environment.” Participants were tasked with moving through all 12 items, which would convey they were able to navigate all areas, engage with features, and customize their avatars. After usability testing, we conducted beta testing, which comprised several questions to assess: (1) avatar customization, (2) functionality and features, (3) quest content, and (4) educational health content. We also asked for any additional feedback for tailoring the virtual environment content before the initiation of the clinical trial. In phase 3, we will test the new content that is embedded and assess outcomes on usability and acceptability.

**Virtual environment features that will be accessible to individuals enrolled in the clinical trial.**

**Features and key activity examples**

- Avatar
  Customize avatar
  Engage anonymously with other participants
- Quests
  Build knowledge about chronic illness prevention
  Test knowledge
  Earn tickets to customize avatar
- Districts
  Lobby (pharmacy and bookstore 1)
  Plaza (fitness center, clothing store, and bookstore 2)
  Food court (restaurant, grocery, and farmers market)
  Reflection garden (tranquil area)
- Nurse-led health sessions (not part of phases 1 and 2)
  Virtual or video presentations focusing on heart health
- Technical support (not part of phases 1 and 2)
  Instructional guidebook on virtual environment use
  Videos to enhance the use of the virtual environment
  Web-based newsletter

### Data Analysis

Qualitative data were analyzed using content analysis in NVivo (version 12; Lumivero). Content analysis is an established set of stepwise techniques used in qualitative research to identify, code, and describe themes and then assign themes to open-ended narratives. In qualitative research, data are contextual and focused on maximizing the understanding of the phenomenon under study and not necessarily on a fixed sample size. A total of 3 coders analyzed the data (SRR, Lauren Chin, and Maame-Owusua Boateng), and discrepancies were resolved through discussion until a consensus was reached. Data saturation was achieved after 15 phase-1 participants were interviewed, and there were no new emerging data [41,42]. In phase-2 usability testing, metrics of content navigation and the duration of time to completion were tracked using the 12-item checklist. Beta-testing qualitative data were pooled, and common themes were assigned thematic categories to contextualize the findings using representative quotes.

### Measures of Rigor for Qualitative Data

Reliability was demonstrated by providing a detailed description and rationale for the study purpose, participant sampling approach, and role of the researchers and study team [43]. Dependability was ensured through using consistent approaches to data collection, such as the use of a prespecified interview guide [44]. Validity was assessed by checking for the accuracy or credibility of the findings through member checking and peer debriefing [43,45]. Debriefings were held with the study team following data collection to strengthen the validity of data interpretations and not leave them subject to recall [43]. Transferability [44] of our study findings may be applicable to diverse and marginalized populations who are at a risk of comorbid conditions. Standards for Reporting Qualitative Research was used to report findings [46].

## Results

### Sample Characteristics

We collected qualitative data about health concerns outside of HIV from 11 participants who are clients at the community-based organizations. Their characteristics are presented in Table 1.

On average, participants reported having been diagnosed with HIV for 5.8 (SD 6.62) years with the range of years spanning from 1 to 21 years. The study participants with HIV reflect the demographic characteristics of individuals who have been traditionally underrepresented in clinical research and disproportionately face significant health disparities.

Of the 15 participants, 4 (27%) were HIV care experts with clinical and community health backgrounds: 1 holistic advanced practice nurse practitioner with a master’s degree and 5 years of experience, 1 access-to-care specialist with a bachelor’s degree and 5 years of experience, 1 community health educator with a master’s degree and 15 years of experience, and 1 HIV senior program director with a bachelor’s degree and 6 years of experience. All 4 HIV care experts identified as being from an ethnic or racial minoritized background. These individuals were highly trusted by participants. Their engagement during phases 1 and 2 facilitated open lines of communication from our participants with HIV, which added to the quality of the data.

The phase-2 sample consisted of 10 participants, which aligned with published recommendations for conducting user testing [47,48]. Overall, 45% (5/11) of client participants with HIV who participated in phase-1 qualitative interviews also participated in phase 2. Educational attainment ranged from 11th grade to a bachelor’s degree.

In phase 2, we enrolled 5 HIV care experts who had backgrounds in medicine and community health. There was an overlap where 1 HIV care expert also participated in the phase-1 qualitative interview. HIV care experts had completed a college level education ranging from an associate’s degree to a Doctor of Medicine degree.

**Table 1:** Phase 1 qualitative interview participant characteristics (n=11)^a^.

Characteristics		Values
**Race and ethnicity, n (%)**		
	African American (non-Hispanic or Latinx)	5 (45)
	Latinx	4 (36)
	Mixed race ethnicity or biracial	2 (18)
Age (y), mean (SD)		32.27 (3.04)
**Highest level of education, n (%)**		
	Master’s degree	—^b^
	Bachelor’s degree	2 (18)
	Some college or associate’s degree	2 (18)
	HS^c^ diploma or GED^d^	7 (64)

^a^HIV care experts (n=4) were not included in the table due to the small sample size. Their characteristics are narrated in the *Results* section.

^b^Not applicable.

^c^HS: high school.

^d^GED: General Educational Development.

### Phase 1: Focus Group and Semistructured Interviews

#### Overview

Qualitative data collection for each interview took 60 to 90 minutes. A total of 9 semistructured interviews and 1 focus group were conducted with 6 participants. Four (67%) of the 6 participants were HIV care experts with clinical and community health backgrounds, and 2 (33%) were client participants with HIV who were receiving services from the community-based organizations. Before the focus group, participants requested a program manager be a part of the group because some participants with HIV were new to participating in research. The program manager contributed ease during the group, which resulted in a robust discussion about HIV comorbidities of concern. Phase 1 resulted in a total of 84 pages of transcribed data.

#### Theme 1: Mixed Perceptions of Health and HIV

Overall, most participants rated their health as “decent” to “really healthy.” They conveyed being very in tune with their health issues and that having a diagnosis of HIV was really the catalyst for being more cognizant about their health behaviors. Of the 11 client participants, 7 (63%) were diagnosed with HIV in the last 5 years and had mixed perceptions regarding how they perceived their overall health. Some of the sentiments conveyed about health perceptions were due to the effects of acquiring HIV during the COVID-19 pandemic (Textbox 2).

Of the 4 HIV care experts, 3 conveyed that mixed perceptions of health-related behaviors (Textbox 3) result from HIV diagnosis in newly diagnosed young people when compared to people who have had HIV for an extended duration.

**Sentiments conveyed by participants about health perceptions.**

**Sentiments**

- “I think me testing positive...being diagnosed with HIV...have helped me open up my mind and tell myself now it’s time to get your health in order...not just your physical health, but your mental health as well...I’ve changed my diet trying to eat healthier, drink healthier, trying to exercise more to help with my physical health, my mental health. Sometimes depending on the weather, instead of taking transportation somewhere I walk just to get a little bit of exercise; or when the weather is warm out, I’ll bring out my bike and I’ll go bike riding around the city just for the exercise” (Pseudonym: Eric).
- “...overall, I feel pretty healthy...I want to live a good long healthy life with the least amount of complications as possible. So yes, if there is ever something, I go to my doctor for, and he’s like, hey, you need to fix this – I’m already on it. For example, like for vitamin D, I think it was my vitamin D, it was really low, and I said okay. Right after the visit, I went right to the store bought those vitamins, started taking those. Three months later, we did my blood work, he’s like, oh, you’re good. I said, thanks” (Pseudonym: Roman).
- “I got diagnosed [with HIV] during the beginning of COVID, like in March...It’s been a process, so to speak, because I would’ve expected for me to be a ho [promiscuous] and then get it [acquire HIV]. But I trusted somebody, you know, that was supposed to be mine and he fell in love with somebody else that was positive. To even know that he got [HIV] positive, and yeah, that’s what, to me, the whole world went...you know issues. Because, I have trust issues already as a person, you know. People show their true colors. It’s common for people to have trust issues after you go through a very traumatic situation” (Pseudonym: Arai).
- “My diagnosis was actually August of 2020, so almost two years. It was a shock. This is on the period of now COVID, and then this happening...This has been a journey in that...from receiving my diagnosis to now has been...as a person also of color, trying to find the right physician that has listened to my needs and my concerns. Living with a compromised immune system especially because of everything, post-diagnosis...it’s been one of those things where I have had to...I’ve had...I felt dismissed in the beginning. I’ve had some concerns as related to other health issues related to diagnosis. So, I’ve had it where some providers have thought I was being a little overly anxious, so they dismissed my concerns or they spoke down to me. At the time until I spoke to other friends, I would have stayed [in care with the same provider] with it because part of it were issues were...maybe financially or economic reasons...I just didn’t have the access to get a better physician. So, I’m stuck with what was familiar with even when I didn’t feel like most of my concerns and medical needs were met. It took some time to navigate that and go out of my comfort or familiarity and then seek out providers who assess my needs. Even if they were a little bit further geographically than I was used to, that overall my experience with them would be a little bit better than what I was already getting” (Pseudonym: Zoe).

**HIV care experts’ selected utterances about clients’ health-related behaviors.**

**Theme 1: mixed perceptions of health and HIV**

- “...people who are living with HIV tend to be more adherent to taking their medications, you know, as prescribed, simply because they want to achieve undetectable viral load. And in addition, in my opinion, they tend to be a little more healthier than people who are not living with HIV” (HIV care expert pseudonym: Tyler).
- “...people that have been already living with HIV for many, many years, they are more conscious about how to take care about themselves, the ways of getting HIV or any other comorbidities, how to take care of themselves. But, I am not very sure that evolves with young people to take care of their issues, their conditions in the same way even though we have more information right now, and more access to treatment. I think that the attitude is a little bit different” (HIV care expert pseudonym: Lloyd).
- “I’ve noticed that over my experience with patients, people with HIV is that there are ebbs and flows, and it depends on where they are in their life and how the perceive their HIV. I do notice that younger folks sometimes think about it like, I have to take this from now until forever for my HIV, and it becomes overwhelming for them at times. I do find that the ones who are diagnosed years ago tend to be more stabilized with their ideas and their thoughts about HIV, and being able to keep them adherent to medication and so forth” (HIV care expert pseudonym: Eric).

#### Theme 2: High Risk for Comorbid Conditions

Participants with HIV perceived that they were susceptible to other illnesses as a result of having HIV, stemming from environmental conditions, such as diabetes and asthma. They spoke about lung function and the effects of the weather on their breathing. CVD and CVD risk factors were consistently expressed concerns in relation to HIV and health, including hypertension, myocardial infarction, stroke, and diabetes. Additional well-described comorbidities of HIV, such as kidney and liver diseases, were also identified by participants as important. Cancer (prostate, colon, and others) was a common concern among participants. Participants with HIV also voiced concerns about mental health conditions. Participants conveyed the following:

I heard that sometimes complications can come with, you know living with HIV, like cancer and other health problems that can come up, that I’m not really...I’m not aware of all of them. But I don’t know; cancer is just something that I thought that could be. And especially too, with heart problems or you know, different things like that...Heart attacks, strokes, palpitations, all kinds of stuff.

Pseudonym: Kori

I have high blood pressure, so I take high blood pressure medicine. I also have asthma, so I usually have a pump just in case. That’s pretty much it.

Pseudonym: Ralph

Cancer is a concern for me because it runs in my family. It’s something that’s kind of, I guess is hereditary. We’ve lost a lot of family members due to that. So, for people who probably have hereditary illnesses or diseases that run in their family, that should be of concern to them.

Pseudonym: Omar

I don’t know why, but colon cancer, and that’s not it, because I remember hearing someone talk about it, and I was just like, this can really happen! I never knew that.

Pseudonym: Roman

The HIV care experts described chronic diseases and mental health conditions that were affecting participants (Textbox 4), such as anxiety and depression due to the stress of living with HIV and, for some, HIV comorbidities.

**HIV care experts’ selected utterances about their clients’ risk for comorbid conditions.**

**Theme 2: high risk for comorbid conditions**

- “I’ve dealt with a lot of diabetes and hypertension dominating those areas. And, I deal with a lot of patients who are HIV positive and diabetes, and like the secondary factor that is a big concern to them...They haven’t learned yet...most of the people say that they’ve not learned what are the best options, as far as nutrition, to take care of their diabetes on top of their HIV. So, they’re focused on what they should or should not be eating ” (HIV care expert pseudonym: Eric).
- “...but another health issue that still didn’t come out. And it’s about liver disease. The population that I work with that are mostly over 45 to 50-years old, this sort of thing has developed. And, in the different part of the body or even cirrhosis, even those these people do not drink any alcohol, okay. So, I feel that it’s another point to keep in mind” (HIV care expert pseudonym: Lloyd).

**Theme 2.1: high risk for mental health conditions**

- “I know we’re talking a lot about more physical, although I do think depression is physical too. But, more physical illnesses, but depression is probably the, really, most common one I’ve heard about, you know, being either diagnosed with depression or anxiety disorder” –(HIV care expert pseudonym: Eric).
- “I work with people who become, at least clinically depressed after their diagnoses. I was going to say that earlier as well. So, there may not actually be like a physical thing, but it certainly can develop into a physical thing, as someone falls into, I guess, a deep depression. Or, like they have these drug induced psychosis and stuff like that, which is quite possible for an HIV induced psychosis as well, you know, because you were recently, you know, diagnosed, that can kick up some things in that person that may have not necessarily been focused on earlier or identified earlier” (HIV care expert pseudonym: Tyler).

#### Theme 3: Virtual Environment Features

We tested out features of the virtual environment, such as avatar customization, quests, and navigation to various districts (Textbox 1). There was excitement around the privacy and anonymity of the platform and the customization of the skin tone, hair color, and clothing of the avatar. Participants were excited about the potential of meeting other participants with HIV who identified as gay or bisexual. Information within the virtual environment was deemed interesting, engaging, and supportive of their identity. Participants commented about naming the bookstore after Marsha P Johnson and the representation of pictures and content that validated race and ethnicity. Overall, the accessibility of the virtual environment to ethnic and racial sexual minority men with HIV, who have not had prior exposure to clinical trials addressing heart disease prevention, was deemed as appealing. The participants’ views are listed in Textbox 5.

HIV care experts had favorable sentiments about the virtual environment. They focused on nutrition, avatar diversity, engagement with others, and pushing the envelope to extend the virtual environment to new areas in preventive screenings (Textbox 6).

**Participants’ views about virtual environment features.**

**Participants’ views**

- “I don’t even think I could add anything else. I think just within itself I feel like it would be amazing because it’s so different. Say maybe somebody wants information or wants to meet other people to discuss things, sometimes it can be nerve wracking or sometimes people aren’t ready to really put themselves out there like that. So, I think with having the avatar and having different spaces, like the library and the relaxation area is good because you could be who you would want to be with the avatar. So, you’re comfortable to really put yourself out there. I think the concept is really good because some people aren’t as up front. It’s like they still get to maintain their sense of privacy, but also get to interact with other people and get more knowledge and stuff about things. Yes, I think it’s great the way it is” (Pseudonym: Ralph).
- “I think that [privacy and anonymity] is important for the simple fact that that people do enjoy their privacy. I would say that’s great, because I'm someone who plays online a lot, and I never am giving out my real name. So, it’s always, this is my gamer tag, and if you want to chat, we can talk on this court where it makes things more private. You don’t have my number, but you can message me on there” (Pseudonym: Roman).
- “I would love that [to use a virtual environment for heart disease prevention], and honestly, for me, like I said, I’m very much a hands-on person. I love the idea of the experiencing new things. So, you had me at, you know, the start of the [virtual environment platform] pictures” (Pseudonym: Arai).
- “...I think that giving tips to us, how to take care of ourselves, better care of ourselves to prevent certain illnesses. I think it should be information about the type of foods to eat, you know vitamins, different tips of how to keep ourselves, you know, healthy” (Pseudonym: Kori).

**HIV care experts’ feedback on beta testing the virtual environment.**

**Feedback**

- “I liked the avatar. I liked how it was easy to change to whether I wanted to be male or female. Skin tone is important. If I wanted to wear something short, something long, it gave the options. So, it wasn’t limited to just I have to be a girl or have to be a boy...And I think that the areas are very colorful. The Reflection area, it looked like a Tiki bar. So it makes you want to meet with people. So it’s a good, it’s visually appealing to say; hey, let’s meet here...Some people are very uncomfortable talking about health messages or their own personal. But if you have an avatar and your avatar can ask questions, or a trivia questions pops up and you're in a group, and they’re now competing with another group, it may be better to get messages out” (HIV care expert pseudonym: Emma).
- “...I definitely liked the areas. I think all the areas was nice. I think the game was well-developed. However, I think maybe the avatars should be maybe a little more reflective of the people using the games. Also, I think that...And I know that you said that this is something that you guys are working on. But changing the menu options and the pharmacy things, the things that people actually use...I think it’s definitely something that we would use with our clients. It’s easy to navigate. The information is there. I mean I work with a lot of people who are undocumented. They have a hard time understanding English. The visual aspect of it is great. It is also something that we can use to keep our clients on track. So if we were able to customize it, I would customize it to questions that reflect the communities that we serve. Like, have you been taking your medication in the past five days?” (HIV care expert pseudonym: Sequan)
- “For example, talking about aging with HIV. How does that look? The other comorbidities that can accompany HIV as well. As we get older, we have so many things that can...We are not as potent as we used to be when we were younger. So we are coming with other comorbidities. And plus, HIV adds into that as well. So how does that look? So what are the other problems that you may have? Also the screenings, what screenings are recommended based on particular times of your life? For example, Are the males doing their colonoscopy on time? It’s recommended to do it at 45 now. It used to be 50. Are you receiving some vaccines? For example, you have Shingrix that protects you against Shingles. Will you have to take that a second time? And you have vaccines against pneumonia, and flu shots. And also, cognition. As we get older, you can have cases of dementia. You can have isolation. How do you meet other people of your age? How do you socialize? There are other things that can...I mean if you want to really push it to the limit, there are so many things that you can identify that can also make a difference” (HIV care expert pseudonym: Mario).
- “I think that’s a good use for it [the virtual environment], just being able to look at the calories and the ingredients in certain food items and such. But also, what I think my favorite part was the gym and the health tips. So like in terms of basic, how much water intake you should have in a day, and information walking. I think one of the things it talked about was how to select a shoe and how to do things like that, for exercise. These are questions that people don’t think about until they have to think about it. And so going there and finding that information I think is helpful” (HIV care expert pseudonym: Kayla).
- “So by adding those varieties of cuisine, for example, like Italian, Caribbean, it’s a way you’re helping the person to make a different choice, if they ever get a chance in the real life. For example, there is a food that you might not know about. And by presenting it to you in the virtual, it’s all right. I mean they might be more willing to try this. And also if you used to hear something about like a food, now that you have the facts, would you want to try it? So I think that’s going to be pretty helpful” (HIV care expert pseudonym: Porter).

### Phase 2: Usability and Beta Testing

**Figure 1:**
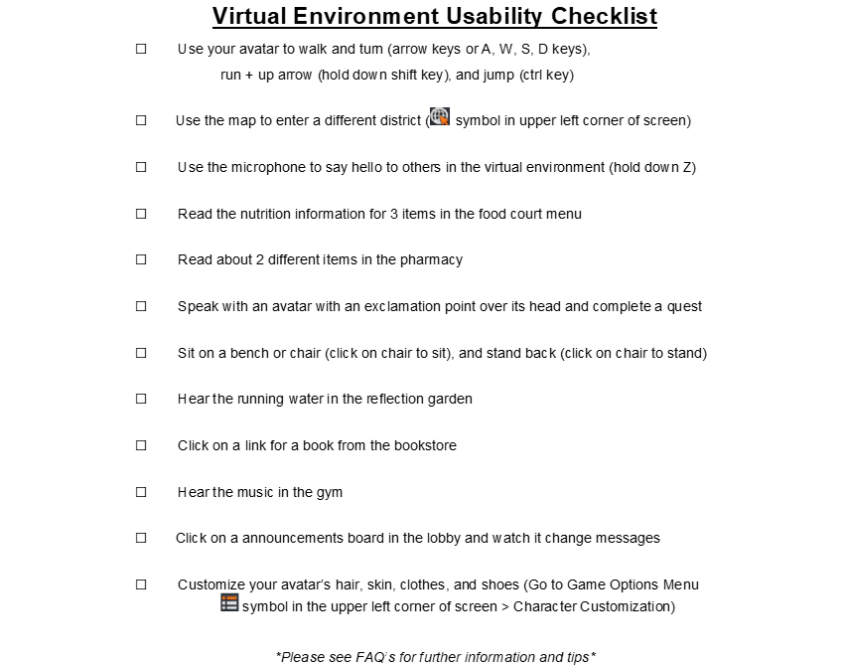
12-item usability checklist. FAQ: frequently asked question.

All 10 participants were able to complete all 12 action items on the checklist (Figure 1).

**Figure 2:**
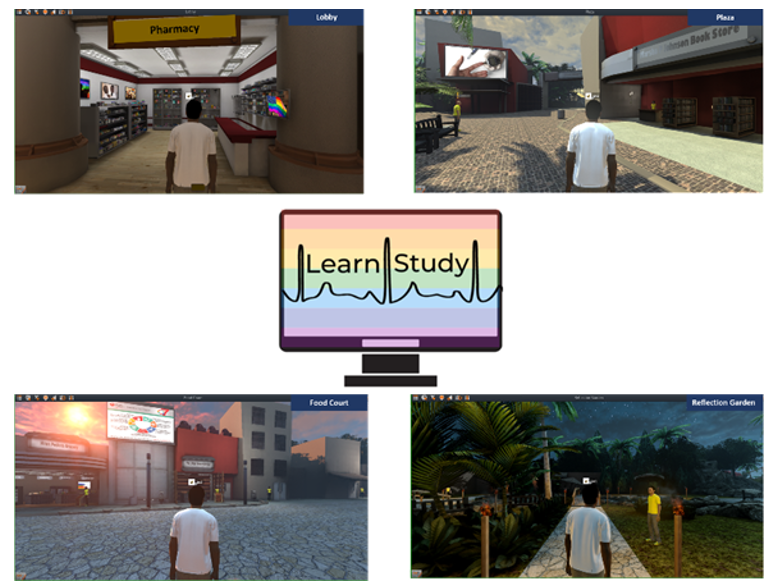
The LEARN virtual environment's 4 districts (lobby, plaza, food court, and reflection garden).

The reflection garden was the most enjoyed area by participants because it was the most serene. The fitness center received the most feedback, especially that it needs more equipment to become more realistic. The duration of time to completion of the checklist ranged from 30 to 60 minutes. Those with more technology experience were able to navigate the checklist faster. Information within the virtual environment was deemed interesting, engaging, and supportive of their identity. Participants commented about naming the bookstore after Marsha P Johnson and the representation of pictures and content that validated race and ethnicity (Figure 2). Overall, feedback from the usability and beta testing of the virtual environment was favorable but also contained constructive feedback for representation and diversity.

Selected utterances from the beta testers are presented in Textbox 7.

**Selected quotes obtained during best testing.**

**Selected quotes**

- “I think the names [of the stores and restaurants] are really creative. And I like how some of them pay homage [to historical lesbian, gay, bisexual, transgender, queer, and others (LGBTQ+) leaders] I think you guys can get creative with the graphics on these graphics here, because you’ve got a bookstore called the Marsha B. Johnson Bookstore. So it’d be nice if you could have maybe pictures of her on the slides, or historical figures on the slides. That would be cool. Yeah. That’s pretty much it. I would just add that” (Pseudonym: Justin).
- “Gay’s Anatomy! I’m dying. I’m so down with this game. This is really cute. Okay...Marsha P. Johnson Bookstore? Marsha P? Come on, honey. Stone wall and all, girl!...But the avatars, they need to be able to customize the body, too. Like make them bigger, if they want them to be bigger. Or make them skinnier if they want them to be skinnier. Because some people might come on something like this not just for the information, but like I said, to meet other people and maybe talk to other people. And I feel like they should be able to emulate themselves in this world like however they want” (Pseudonym: Beta4).
- “I would change the texture of the hair [on the avatars] and have more options of the shoes and colors of pants. His [the avatar’s] hair is more like one of them Backstreet Boys hair. What about curly? What about afro? What about braids” (Pseudonym: Felix).
- “I would be more interested in the trivia, simply because I can learn something new. And it’s easy to figure out, because I get explanations on why I’m right or wrong. And if I have questions about something, I can always go to a different section and be informed about my questions.” (Pseudonym: Silver).

## Discussion

### Principal Findings

The purpose of this study was to explore perceptions of HIV-related comorbidities and assess the usability of a virtual environment for CVD prevention education in Black and Latinx sexual minority men with HIV. We found that perceptions of health among participants were mixed and based on life context. Those who perceived their health in a positive light were motivated to practice enhanced health behaviors, such as enhancing physical activity, medication adherence, and following clinician health recommendations, as a result of acquiring HIV. Negative sentiments toward health perceptions were reported due to personal relationships, being diagnosed with HIV during the COVID-19 pandemic, and socioeconomic determinants that limited accessibility to lesbian, gay, bisexual, transgender, queer, and others (LGBTQ+) health care specialists. Cancer was identified as a predominant illness of concern. There is an increased risk of cancers, other than AIDS-defining illnesses, among people with HIV, and concern may be due to reported heredity factors as well as potential risk due to shared cancer-related risk factors. In a recent study by Bell et al [49], CVD was associated with a higher incidence of cancer, especially in persons with atherosclerotic CVD, which carries significant implications for mitigating barriers to preventive health screenings and raises considerations for cancer and multimorbidity in Black and Latinx sexual minority men with HIV.

With regard to the virtual environment, almost all phase-1 client participants with HIV (10/11, 91%) were enthusiastic. We believe that hesitancy in the remaining participant was related to not having prior access to or experience with evolving technologies and unfamiliarity with the premise of using a virtual environment. Participants with HIV were particularly open to the use of a virtual environment because they would assume the identity of an avatar, with a pseudonym, and would meet others who had shared characteristics and health goals. Participants were pleased about the potential to learn with others who were a part of the study, which is an important consideration in behavioral interventions, as individuals build community around shared life experiences. We found that the participants were interested and found the content desirable because we named the bookstore after Marsha P Johnson (a Black transgender woman who was a pioneer and activist for gay and transgender rights) and included positive visualizations of ethnic and racial sexual minority men, queer love, and pride. This was important to validate and affirm the existence of the participants. These types of visualizations are uncommon in public areas in the United States and are banned in certain states. Providing a virtual environment that celebrates ethnic and racial LGBTQ+ individuals and legacy activists was deemed as acceptable and wanted.

HIV care experts are trusted individuals who work with the community and have keen insights into their needs, challenges, and opportunities to enhance cardiovascular health. Their lived and professional experiences working with ethnic, racial, and sexual minoritized individuals enhanced our understanding about the intersectionality of race, ethnicity, HIV serostatus, and CVD risk in relation to community needs.

The prevention of comorbidities in persons with HIV is complicated due to historic and ongoing barriers to health equity in health care and research [20] that disproportionately target racial, ethnic, sexual minority populations and other marginalized populations [11]. CVD prevention is also challenging because individuals are required to become proficient in a variety of lifestyle behaviors to enhance cardiovascular health and incorporate these into their everyday lives [50]. Our study findings was favorable toward the use of a virtual environment for CVD prevention education in ethnic and racial sexual minority men with HIV. These findings are in alignment with a 2019 survey conducted by the AARP formerly known as the American Association of Retired Persons, which suggested that 50% of African American and 42% of Latino persons aged >50 years played video games [51,52]. The 2 prominent reasons for engaging in video game play were entertainment and the maintenance of mental sharpness [51,52]. Moreover, other studies have used virtual environments (defined as 3D computerized environments with avatars) for risk reduction in adolescents [53,54], diabetes self-management [37,55], and cardiac rehabilitation [56]; however, none have focused on CVD prevention in persons with HIV. Recommendations from a systematic review identified the need for future research on gamification for CVD prevention to include (1) larger sample sizes, (2) experimental designs, (3) objective measures, and (4) behavior change frameworks [57]. Our qualitative study, used to inform our future clinical trial [33], directly responds to the need to develop novel, widely applicable strategies to address cardiovascular health equity using a virtual environment for individuals from a variety of minoritized racial and ethnic groups with HIV, particularly those who might otherwise lack access to clinical trials research**.**

### Limitations

First, although our sample size was in alignment with qualitative guidelines, small sample sizes limit generalizability. However, the diverse participant sample and inclusion of HIV care experts provided insights into perceived CVD and other comorbid risks and the potential for the use of a virtual environment for CVD prevention education. This type of data has not been captured in previous studies from an exclusive participant sample of economically marginalized, ethnic and racial sexual minority men with HIV. Second, using qualitative methods, we were unable to demonstrate causality [58]. However, we followed established criteria for ensuring rigor during data collection [58] and obtained >80 pages of qualitative data. A strength of qualitative data collection was the ability to gain high-quality contextual insights and details into HIV comorbidities of concern and assess the usability of and verbal reactions about the virtual environment, which would not be obtainable using survey methods. Third, recruitment bias is a potential limitation. We minimized recruitment bias by adhering to our prespecified inclusion criteria.

### Conclusions

We identified several comorbid conditions of concern, and findings carry significant implications for mitigating barriers to preventive health screenings in Black and Latinx sexual minority men with HIV, given the shared risk factors between HIV and related comorbidities. Highly rated aspects of the virtual environment were anonymity, the ability to meet others with HIV who identify as gay or bisexual, validating LGBTQ+ inclusive images and content, and accessibility to CVD prevention education. Critical end-user feedback from beta testing suggested more options for avatar customization in skin, hair, and body representation. We will apply these recommendations and others in phase 3 to formally test the virtual environment in the subsequent clinical trial.

## Appendix

Multimedia Appendix 1 Standards for Reporting Qualitative Research (SRQR) checklist.

## Abbreviations

CVD: cardiovascular disease
HRV: heart rate variability
LGBTQ+: lesbian, gay, bisexual, transgender, queer, and others
LIVE: Learning in a Virtual Environment

## Data Availability

The data sets generated during and analyzed during this study are not publicly available or available for sharing given the small sample size and even the combination of indirect identifiers could possibly identify a participant.
